# Radiologically Measured Renal Pelvis Urine Density Is Associated with Severe Infectious Complications After Ureteroscopy for Ureteral Stones: A Post Hoc Analysis of a Multicenter Prospective Study

**DOI:** 10.1016/j.euros.2025.09.002

**Published:** 2025-09-22

**Authors:** Zichuan Yao, Po-Hua Lin, Kaiqi Zhang, Hongliang Ji, Shiyu Song, Fei Wu, Hanfeng Xu, Zhen Liu

**Affiliations:** a Department of Urology, Shengjing Hospital of China Medical University, Shenyang, China; b The Fifth Hospital of Liaoyang City, Liaoyang, China; c Luhe Hospital of Yingkou City, Yingkou, China; d Department of Radiology, Affiliated Zhongshan Hospital of Dalian University, Dalian, China; e Department of Urology, The First Affiliated Hospital, Hengyang Medical School, University of South China, Hengyang, Hunan, China; f Department of Urology, The People’s Hospital of Liaoning Province, The People’s Hospital of China Medical University, Shenyang, China

**Keywords:** Hounsfield unit, Renal pelvis density, Ureteroscopic lithotripsy, Infectious complication, Urosepsis

## Abstract

**Background and objective:**

Finding reliable and easy-to-obtain indicators of severe infectious complications after ureteroscopy represents a major clinical need. This study aimed to assess the association between Hounsfield units (HUs) in renal pelvis urine and severe infectious complications in patients with ureteral stones and asymptomatic hydronephrosis following ureteroscopy.

**Methods:**

In this post hoc analysis of a multicenter prospective trial, renal pelvis urine density (RPUD) was measured via non–contrast-enhanced computed tomography. Severe complications (septic shock, urosepsis, and systemic inflammatory response syndrome) were recorded. Odds ratios (ORs) with 95% confidence intervals (CIs) were calculated by multivariable logistic regression.

**Key findings and limitations:**

Among 601 patients, 9.5% (57/601) developed severe infectious complications. After adjustment, each one HU unit increase in RPUD was associated with 54% higher odds of severe infectious complications (adjusted OR = 1.54; 95% CI: 1.37–1.73; *p* < 0.001). Restricted cubic splines confirmed a linear relationship (nonlinear *p* = 0.2). Gender (female vs male, adjusted OR = 3.55; 95% CI: 1.57–8.02; *p* = 0.002), hydronephrosis grade (G3 or G4 vs G1 or G2, adjusted OR = 4.86; 95% CI: 1.89–12.5; *p* = 0.001), and mean stone density (lower vs higher, adjusted OR = 0.996; 95% CI: 0.994–0.997; *p* < 0.001) were also independently associated with severe infectious complications.

**Conclusions and clinical implications:**

This study demonstrates a positive linear association between renal pelvis urine HU values and severe infectious complications after ureteroscopy, independent of comprehensive patient and stone characteristics.

**Patient summary:**

We found that higher urine density in the renal pelvis (measured by a computed tomographic scan) is significantly associated with the incidence of severe infectious complications after ureteroscopy for ureteral stones.

## Take Home Message

This multicenter prospective study establishes, for the first time, a significant positive association between elevated renal pelvis urine density (measured in Hounsfield units [HUs]) and severe infectious complications (eg, systemic inflammatory response syndrome, urosepsis, and septic shock) following ureteroscopy in patients with ureteral stones and asymptomatic hydronephrosis. After adjusting for patient and stone characteristics, each 1 HU increase in renal pelvis urine density was associated with a 54% higher risk of complications (adjusted odds ratio [OR] = 1.54; 95% confidence interval [CI]: 1.37–1.73, *p* < 0.001). Additionally, gender (female vs male, adjusted OR = 3.55; 95% CI: 1.57–8.02; *p* = 0.002), hydronephrosis grade (G3 or G4 vs G1 or G2, adjusted OR = 4.86; 95% CI: 1.89–12.5; *p* = 0.001), and mean stone density (lower vs higher, adjusted OR = 0.996; 95% CI: 0.994–0.997; *p* < 0.001) were also independently associated with complications. This study demonstrates a positive linear association between renal pelvis urine HU values and severe infectious complications after ureteroscopy, independent of comprehensive patient and stone characteristics.

## 1 Introduction

Approximately 10–12% of adults will be diagnosed with urolithiasis during their lifetime [1]. Ureteroscopy (URS) is one of the most common treatments for ureteral stones due to its high success rates [2]. However, urosepsis is a serious potential complication of ureteroscopic procedures, posing significant risks such as prolonged hospitalization, intensive care unit admission, and even fatal outcomes [3,4]. A recent meta-analysis of 5597 patients undergoing URS for stone disease reported postoperative urosepsis rates ranging from 0.2% to 17.8%, with a pooled incidence rate of 5.0%. The identified risk factors include preoperative stent placement, preoperative positive urine culture, ischemic heart disease, older age, longer procedure time, and diabetes mellitus [3].

Non–contrast-enhanced computed tomography (NCCT) is routinely performed to evaluate the condition of hydronephrosis and the parameters (position, density, and diameter) of urinary stones. In cases of acutely infected hydronephrosis, NCCT may show signs such as thickened renal pelvic walls, inflammatory changes in the renal parenchyma or perinephric region, and gas-fluid or fluid-fluid levels in the urinary collecting system [5]. However, nonsymptomatic hydronephrosis without specific computed tomography (CT) signs is frequently observed in clinical practice [6,7]. In the early stages of infected hydronephrosis, abnormalities may be absent on NCCT, particularly after lithotripsy procedures, increasing the risk of inappropriate treatment and severe infectious complications.

Previous studies have reported a positive association between the Hounsfield unit (HU) value of renal pelvis urine and pyonephrosis [[8], [9], [10]]. However, the relationship between HU in renal pelvis urine and the risk of serious infectious complications following ureteroscopic lithotripsy has not been investigated thoroughly. A retrospective study of 122 patients with symptomatic hydronephrosis caused by stones or tumors found higher HU values in the sepsis group than in the nonsepsis group after decompression [11]. However, this study included only symptomatic patients, did not address lithotripsy, and lacked adjustment for essential stone parameters such as size and density.

This multicenter prospective cohort study quantifies the association between renal pelvis urine HU values and severe infectious complications (eg, systemic inflammatory response syndrome [SIRS] and urosepsis) in patients with ureteral stones and asymptomatic hydronephrosis following ureteroscopic lithotripsy. By addressing methodological limitations of previous studies, it establishes this relationship using prospective multicenter data.

## 2 Patients and methods

### 2.1 Study design

**Fig. 1:**
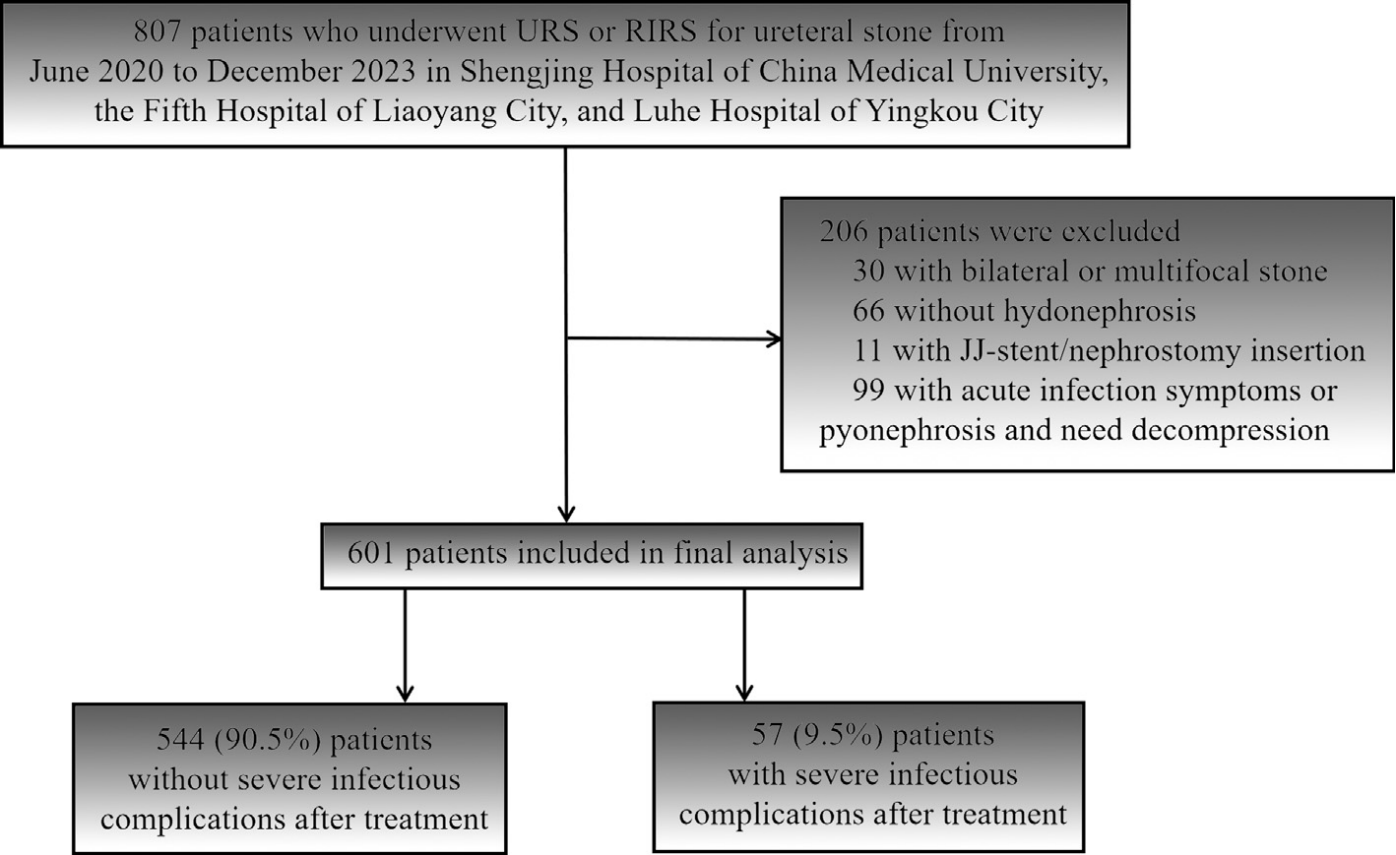
Flowchart of this cohort. RIRS = retrograde intrarenal surgery; URS = ureteroscopy.

This study is a post hoc analysis of a registered prospective multicenter trial, which was designed to compare ureteroscopic lithotripsy and extracorporeal shock wave lithotripsy for ureteral calculi. The current analysis focuses on a subset of 601 patients with ureteral stones and asymptomatic hydronephrosis from the original trial population treated at three participating hospitals from June 2020 to December 2023: Shengjing Hospital of China Medical University, the Fifth Hospital of Liaoyang City, and Luhe Hospital of Yingkou City; see Fig. 1 for details. The Ethics Committee of Shengjing Hospital Affiliated to China Medical University granted ethical approval (2020PS520K). All eligible participants provided written informed consent. The study was registered with the Chinese Clinical Trial Registry (ChiCTR2000033790). The research protocol adhered to the ethical standards set forth in the Declaration of Helsinki (1975).

### 2.2 Inclusion and exclusion criteria

#### 2.2.1 Inclusion criteria

The inclusion criteria were as follows: patients with ureteral stones (including pelvic-ureteral junction stones) of 6–20 mm in size, age ≥18 yr, and patients with nonsymptomatic hydronephrosis (described as G1–4 hydronephrosis with normal preoperative white blood cell count, body temperature, and negative urine culture).

#### 2.2.2 Exclusion criteria

The exclusion criteria were as follows: pyonephrosis, which was confirmed upon the observation of pyuria (purulent debris and sediment) in the urinary collecting system during endoscopic surgery; perirenal abscess or retroperitoneal abscess; positive blood or/and urine culture before surgery; renal insufficiency; prior nephrostomy or double-J (JJ) stent placement; multiple or bilateral ureteral stones; transplanted kidney; solitary kidney; anatomical obstruction distal to the stone; and congenital genitourinary anomalies.

### 2.3 The technique of URS and retrograde intrarenal surgery

All procedures were conducted under either spinal or general anesthesia. An Fr 8/9.5 rigid URS (Richard Wolf, Knittlingen, Germany) was employed commonly for middle or distal ureter stones. Fragmentation was performed using pneumatic and ultrasonic lithotripsy systems (EMS, Nyon, Switzerland) or holmium:yttrium-aluminum garnet (YAG) laser (Lumenis PowerSuite 60w, Yokneam, Israel), with a 200 μm laser fiber through ureteral access sheaths. Fragments were extracted by forceps or nitinol baskets. Stone migration into the renal pelvis was mitigated using antimigration devices (Stone Cone). An Fr 5 JJ stent was placed after URS.

The surgical technique of retrograde intrarenal surgery (RIRS) was performed under general anesthesia in the lithotomy position. For proximal ureteral stones, an Fr 12/14 or Fr 11/13 hydrophilic-coated ureteral access sheath was introduced over a guidewire, following the use of an Fr 8/9.8 rigid ureteroscope (Karl Storz, Tuttlingen, Germany). Subsequently, an Fr 7.5 flexible ureteroscope (URF-P5; Olympus, Tokyo, Japan) or a single-use video endoscope (Guangzhou Ruipai Medical Devices, Guangzhou, China) was inserted. A holmium:YAG laser (Lumenis PowerSuite 60W, Yokneam, Israel) with a 200-µm laser fiber was used for lithotripsy, with energy settings of 1–1.5 J at 10–15 Hz, adjusted according to stone hardness and efficacy. Stone fragments were extracted using forceps or nitinol baskets. Irrigation was performed using a pressure-controlled combination irrigation/suction pump, with pressure settings between 100 and 200 mmHg. In complex cases, an Fr 5 JJ stent was placed after URS and removed approximately 2 wk later.

### 2.4 Regimen(s) of preoperative antibiotic prophylaxis

Intravenous antibiotics were administered to all patients 1 h prior to surgery for prophylactic purposes and were maintained for an additional 24–48 h after the procedure. The choice of antibiotic prophylaxis was tailored to institutional antimicrobial susceptibility (aminopenicillin/beta-lactamase inhibitor, cephalosporin group 2 or 3, levofloxacin, and ciprofloxacin).

### 2.5 Baseline characteristics and follow-up

Proximal ureter stones are located between the upper border of the sacroiliac joint and the pelvic ureteric junction. Middle and distal ureter stones were defined as those between the upper border of sacroiliac junction and the orifice of the ureter in the bladder [12]. The size of the stone was defined by its maximum diameter, as determined by NCCT. The extent of hydronephrosis was assessed using CT and classified into four grades (1–4): the kidney with pelvic dilation only was classified as grade 1 and that accompanied by mild calix dilation was classified as grade 2, the kidney with severe calix dilation was classified as grade 3, and the kidney with calix dilation accompanied by renal parenchyma atrophy was classified as grade 4 [13].

The mean stone density was measured by the mean HUs of NCCT (CT, 3.0 mm/120 kV/200 mA s; GE Healthcare Technologies, Waukesha, WI, USA) in the elliptical region of interest incorporating the largest cross-sectional area of stone without including adjacent soft tissue [14]. Renal pelvis urine density (RPUD) was measured by delineating an elliptical region encompassing the majority of urine within the renal pelvis (excluding adjacent renal parenchyma and calculi) of the affected kidney. The mean HU value was then assessed quantitatively on axial NCCT images. Two experienced urologists performed blinded measurements of each image, and the mean HU value was directly extracted from the PACS system. The intraclass correlation coefficient for HU measurements between the two investigators was 0.877. Specific NCCT signs of infection include a thickened renal pelvic wall and inflammatory alterations in the parenchyma [5]. The time course of NCCT to the operating room was within 1 wk.

Severe infectious complications included SIRS, sepsis, and septic shock, all of which occurred within 1 mo following URS. SIRS included at least two of the following four criteria: (1) body temperature >38 °C or <36 °C, (2) heart rate >90/min, (3) respiratory rate >20 breaths/min or PaCO_2_ <32 mmHg (<4.3 kPa), and (4) leucocyte count >12 000 or <4000 cells/mm^3^, or >10% immature bands [15]. *Urosepsis* was represented by an increase in the sequential (sepsis-related) organ failure assessment score of ≥2 points due to urinary tract infection [16]. *Septic shock* was defined as a vasopressor requirement to maintain a mean arterial pressure of ≥65 mmHg and a serum lactate level of >2 mmol/l (>18 mg/dl) in the absence of hypovolemia [17]. Postprocedural follow-up assessments were conducted during the patient's hospital stay.

### 2.6 Statistical analysis

The normality of continuous variables was assessed using the Kolmogorov-Smirnov test. Normally distributed continuous data were presented as mean ± standard deviation, while non-normally distributed continuous variables were presented as median (interquartile range). Simple (unadjusted) logistic regression models were used to explore the associations between each baseline factor and the outcome. Factors considered clinically important or with p < 0.05 in these unadjusted analyses were then entered into a multivariable binary logistic regression model.

Multivariable binary logistic regression was used to assess the association between RPUD (as a continuous variable, measured in HU) and severe infectious complications after URS. The model was adjusted for clinically relevant confounders, including gender, hydronephrosis grade, mean stone density, body mass index (BMI), and endoscopy type, which either had *p* < 0.05 or were clinical relevant. Results are reported as adjusted ORs with 95% CIs. Restricted cubic splines were used in the fully adjusted model to assess nonlinear relationships.

All statistical analyses were performed using SAS (version 9.4) for Windows (SAS Institute Inc., Cary, NC, USA) and R software (version 4.3.2; https://www.r-project.org/). The R packages “rms,” “ggplot2,” and “splines” were employed in this study. Statistical significance was defined as two-sided *p* < 0.05.

## 3 Results

Finally, 601 patients with ureteral stones were enrolled in this study. The median values for age and BMI among the patients were 48 yr and 23.7 kg/m^2^, respectively. The majority of the patients were male, accounting for 67% of the total population. Of the patients, 9.5% (57/601) developed severe infectious complications following endoscopic surgery. Compared with the nonsevere infectious complication group, patients experiencing severe infectious complications after endoscopic surgery were predominantly female (61% vs 30%, *p* < 0.001), had a higher BMI (27.1 vs 23.4 kg/m^2^, *p* < 0.001), exhibited increased RPUD (16.2 vs 7.7 HU, *p* = 0.001), presented lower stone density (828 vs 1069 HU, *p* < 0.001), and had a higher incidence of severe hydronephrosis (G3/G4, 37% vs 16%, *p* < 0.001), and a higher proportion of these patients underwent URS (54% vs 39%, *p* = 0.022); for further details, refer to Table 1.

**Table 1:** Characteristics and univariate analysis according to infectious status in patients after ureteroscopic lithotripsy

Variables	Total cohort	Noninfectious complications	Infectious complications	*p* value
Number of patients (%)	601 (100)	544 (91)	57 (9.0)	
Renal pelvis urine density				
Mean HU value measured by NCCT	8.5 ± 4.9	7.7 ± 4.1	16.2 ± 5.3	<0.001
Demographic characteristics				
Age (yr)	48 ± 16	48 ± 15	52 ± 19	0.060
Gender (male)	401 (67)	379 (70)	22 (39)	<0.001
BMI (kg/m^2^)	23.7 ± 5.5	23.4 ± 5.4	27.1 ± 5.1	<0.001
Comorbidities				
Hypertension (yes)	101 (17)	96 (18)	5 (8.8)	0.088
Diabetes mellitus (yes)	89 (15)	84 (15)	5 (8.8)	0.18
Coronary heart disease (yes)	40 (6.7)	39 (7.2)	1 (1.8)	0.12
Previous history of urinary stone				0.7
No or spontaneous passage	536 (89)	486 (89)	50 (88)	
SWL/URS/RIRS/PCNL	65 (11)	58 (11)	7 (12)	
Endoscopy type				
RIRS versus URS	359 (60)/242 (40)	333 (61)/211 (39)	26 (46)/31 (54)	0.022
Surgical duration (min)	59.8 ± 10.4	59.9 ± 10.4	59.1 ± 10.1	0.6
Stone characteristics				
Mean stone density (HU)	1046 ± 239	1069 ± 230	828 ± 220	<0.001
Stone side (left)	350 (58)	320 (59)	30 (53)	0.4
Stone location				0.086
Proximal ureter stone	379 (63)	349 (64)	30 (53)	
Middle and distal ureter stone	222 (37)	195 (36)	27 (48)	
Stone size (diameter, mm)	11 ± 3	11 ± 3	11 ± 3	0.6
Hydronephrosis				<0.001
G1 or G2	493 (82)	457 (84)	36 (63)	
G3 or G4	108 (18)	87 (16)	21 (37)	
Specific NCCT signs of infection (yes)	17 (2.8)	14 (2.6)	3 (5.3)	0.2

BMI = body mass index; G = grade; HU = Hounsfield unit; NCCT = non–contrast-enhanced computed tomography; PCNL = percutaneous nephrolithotomy; RIRS = retrograde intrarenal surgery; SWL = shock wave lithotripsy; URS = ureteroscopy.

Continuous variables with a normal distribution are reported as the mean ± standard deviation (SD), non-normal continuous variables are expressed as the median (interquartile range), and categorical variables are reported as the number (percentage). Simple (unadjusted) logistic regression models were used to explore associations between each baseline factor and the outcome. Factors considered clinically important or with *p* < 0.05 in these unadjusted analyses were then entered into a multivariable binary logistic regression model.

**Fig. 2:**
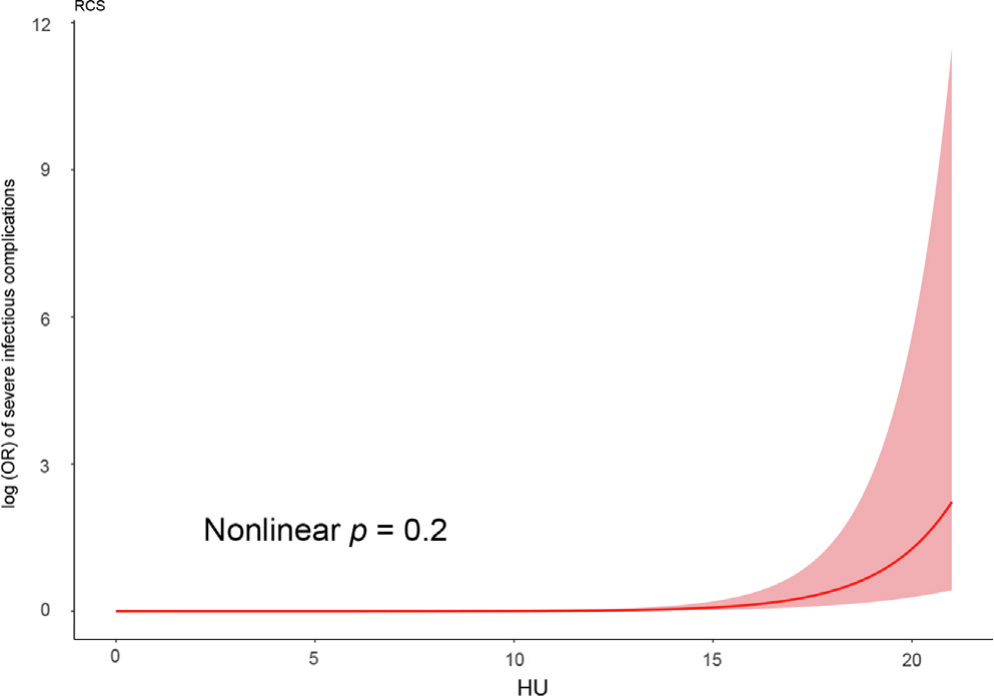
RCS between HU value in renal pelvis urine and severe infectious complications in patients with ureteral stones and nonsymptomatic hydronephrosis after ureteroscopy. HU = Hounsfield unit; OR = odds ratio; RCS = restricted cubic splines.

**Fig. 3:**
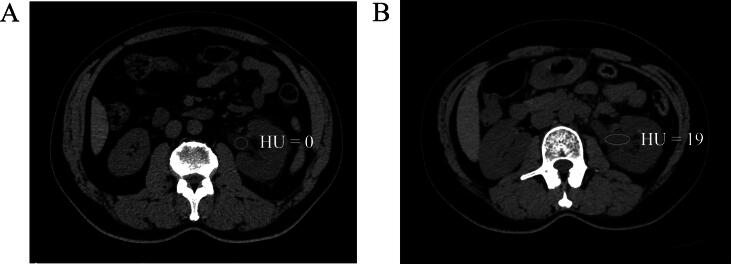
Examples for the measurement of renal pelvis urine by NCCT: (A) an example of the lower value of HU in renal pelvis urine in the patient who was free of severe infectious complications after ureteroscopy and (B) an example of the higher value of HU in renal pelvis urine in the patient who experienced severe infectious complications after ureteroscopy. HU = Hounsfield unit; NCCT = non–contrast-enhanced computed tomography.

Each one-unit increase in RPUD (measured in HU) was associated with a 54% higher risk of severe infectious complications (adjusted OR = 1.54; 95% CI: 1.37–1.73; *p* < 0.001), as determined by multivariable binary logistic regression with adjustment for gender, hydronephrosis grade, mean stone density, BMI, and endoscopy type. Additionally, gender (female vs male, adjusted OR = 3.55; 95% CI: 1.57–8.02; *p* = 0.002), hydronephrosis grade (G3 or G4 vs G1 or G2, adjusted OR = 4.86; 95% CI: 1.89–12.5; *p* = 0.001), and stone density (lower vs higher, adjusted OR = 0.996; 95% CI: 0.994–0.997; *p* < 0.001) were also independently associated with severe infectious complications; see the details in Table 2. Furthermore, the nonlinear association assessed using restricted cubic splines was not statistically significant (nonlinear *p* = 0.2); refer to Fig. 2 for details. Here, we have also provided examples of the CT value measurement for renal pelvis urine; refer to Fig. 3 for details.

**Table 2:** Multivariable binary logistic regression of severe infectious complications after ureteroscopy

Variables	OR	95% CI	*p* value
Renal pelvis urine density (HU)	1.54	1.37, 1.73	<0.001
Gender (female vs male)	3.55	1.57, 8.02	0.002
Hydronephrosis (G3 or G4 vs G1 or G2)	4.86	1.89, 12.5	0.001
Mean stone density (HU)	0.996	0.994, 0.997	<0.001
BMI (kg/m^2^)	1.06	0.975, 1.16	0.14
Endoscopy type (URS vs RIRS)	2.04	0.863, 4.84	0.085

BMI = body mass index; CI = confidence interval; G = grade; HU = Hounsfield unit; OR = odds ratio; RIRS = retrograde intrarenal surgery; URS = ureteroscopy.

The odds ratio and 95% confidence interval were measured through binary logistic regression.

## 4 Discussion

This multicenter prospective cohort study demonstrated a statistically significant positive association between the HU values of renal pelvis urine and the risk of severe infection-related complications, including SIRS and sepsis, in patients with ureteral stones and asymptomatic hydronephrosis following URS.

NCCT is commonly used to assess urinary stone density, but this study is the first to report that higher HU levels in renal pelvis urine are associated with an increased risk of SIRS or sepsis in patients with ureteral stones and asymptomatic hydronephrosis. Supporting this finding, a retrospective study of 122 patients with hydronephrosis (80% stones and 20% tumors) found significantly higher HU values in sepsis cases (12.4 vs 2.7 HU, *p* < 0.001), with HU ≥7.3 independently predicting sepsis (OR = 7.35, 95% CI: 2.56–16.8, *p* < 0.001). These results confirm that renal pelvis HU values can differentiate hydronephrosis from pyonephrosis and predict postoperative sepsis, which aligns with our observations regarding its association with infectious complications in obstructive uropathy [11]. Additionally, previous studies have shown that HU values in renal pelvis urine can predict positive bacterial cultures in patients with hydronephrosis [[8], [9], [10]]. Yuruk et al [10] demonstrated that renal pelvis HU values effectively differentiate pyonephrosis (≥9.21 HU) from hydronephrosis (*p* < 0.001), reinforcing the diagnostic utility of HU measurements in urinary tract infections [10]. A study of 322 patients with upper urinary tract stones and hydronephrosis found significantly higher HU values in the pyonephrosis group than in the hydronephrosis group (14.5 vs 6.4 HU, *p* < 0.001). A multivariable analysis confirmed that HU values were an independent risk factor of pyonephrosis after URS or percutaneous nephrolithotomy (OR = 1.31, *p* = 0.001) [9]. These findings validate NCCT’s utility in quantifying RPUD, aiding earlier diagnosis and appropriate treatment. In this prospective cohort, higher renal pelvis urine HU values demonstrated a significant linear association with severe infectious complications following ureteroscopic lithotripsy (adjusted OR = 1.54; 95% CI: 1.37–1.73; *p* < 0.001), consistent with Caglar et al’s [18] findings (RPUD ≥14 HU predicted postoperative infections in 588 RIRS cases). However, they only recruited patients who were treated with RIRS.

The elevated HU values in asymptomatic hydronephrosis are likely due to increased fluid density caused by infection in the obstructed collecting system, including infected material, cellular debris, and microorganisms [10]. This may explain the association between positive renal pelvis urine cultures and high HU values [8,10].

The study has several limitations. First, its observational design does not establish causality. Second, residual or unmeasured confounding factors may have influenced the results despite adjustments. Third, limited information was provided regarding urinary stone composition. Fourth, while HU measurements were performed by two experienced urologists, the size of the region of interest was not standardized, potentially introducing variability. However, the investigators demonstrated a strong interclass association (0.877) in HU measurements. Fifth, the number of observed outcome events was limited (57 events), which may reduce the statistical power of the study [19]. Future studies with larger sample sizes and more events are needed to confirm these associations.

Despite these limitations, this is the first multicenter prospective study to investigate the relationship between HU measurements in renal pelvis urine and their association with severe infectious complications in patients with ureteral stones and asymptomatic hydronephrosis.

## 5 Conclusions

This study demonstrates a positive linear association between renal pelvis urine HU values and severe infectious complications after URS, independent of comprehensive patient and stone characteristics.

  ***Author contributions*:** Zhen Liu had full access to all the data in the study and takes responsibility for the integrity of the data and the accuracy of the data analysis.

  *Study concept and design*: Liu, Xu, Wu.

*Acquisition of data*: Yao, Lin, Zhang, Ji, Song, Wu, Xu, Liu.

*Analysis and interpretation of data*: Yao, Lin, Zhang, Ji, Song, Wu, Xu, Liu.

*Drafting of the manuscript*: Yao, Lin, Zhang.

*Critical revision of the manuscript for important intellectual content*: Liu, Xu, Wu.

*Statistical analysis*: Yao, Lin, Zhang.

*Obtaining funding*: Liu.

*Administrative, technical, or material support*: Liu, Xu, Wu.

*Supervision*: Liu, Xu, Wu.

*Other*: None.

  ***Financial disclosures:*** Zhen Liu certifies that all conflicts of interest, including specific financial interests and relationships and affiliations relevant to the subject matter or materials discussed in the manuscript (eg, employment/affiliation, grants or funding, consultancies, honoraria, stock ownership or options, expert testimony, royalties, or patents filed, received, or pending), are the following: None.

  ***Funding/Support and role of the sponsor*:** This study was financially supported by Liaoning Provincial Natural Science Foundation Program (project number: 2023-MS-051) by Zhen Liu and Science and Technology Program of Shenyang city (Project Number: 22-321-33-47) by Zhen Liu. These sponsors had no role in the study design; collection, analysis, or interpretation of data; writing of the report; or decision to submit the article for publication.

  ***Acknowledgments*:** We give special thanks to all the colleagues at the Department of Urology for their help and support. We thank International Science Editing (http://www.internationalscienceediting.com) for editing this manuscript. The authors would like to thank all the study participants.

  ***Ethics statement*:** Ethical approval (2020PS520K) was provided by the Ethics Committee of Shengjing Hospital Affiliated China Medical University. Informed consent was obtained from all eligible patients. The clinical research registry UIN is ChiCTR2000033790. The study protocol conformed to the ethical guidelines of the 1975 Declaration of Helsinki.
